# Somerset County Lunatic Asylum

**Published:** 1850-04-01

**Authors:** 


### SOMERSET COUNTY LUNATIC ASYLUM.

We feel much pleasure in directing the attention of our readers to the beautiful lithographic representation of this asylum, which accompanies the present number of our journal. The architect is Mr. Moffatt, of Spring Gardens, a gentleman destined to earn great distinction in his profession. We had prepared an analysis of the first report of this institution, in conjunction with several other reports of British Lunatic Asylums which we have been favoured with, but we are reluctantly compelled to defer its publication until our next number.

